# Preoperative Laboratory, Imaging, and Risk Assessment Before Elective Colorectal Cancer Resection: What the Clinician Must Remember

**DOI:** 10.3390/jcm15187061

**Published:** 2026-09-11

**Authors:** Sophia Tsokkou, Paraskevi Chatzikomnitsa, Menelaos Papakonstantinou, Areti Danai Gkaitatzi, Eftychia Liampou, Georgia Kolympa, Antonios Fantakis, Evdokia Toutziari, Dimitrios Giakoustidis, Theodora Papamitsou, Vasileios N. Papadopoulos, Alexandros Giakoustidis

**Affiliations:** 1 First Department of Surgery, General Hospital “Papageorgiou”, School of Medicine, Faculty of Health Sciences, Aristotle University of Thessaloniki, 54124 Thessaloniki, Greece; voula.hatzikomnitsa@gmail.com (P.C.); menelaospap.md@gmail.com (M.P.); aretidanaegtz24@gmail.com (A.D.G.); liampouef7@yahoo.gr (E.L.); georgettekolympa@gmail.com (G.K.); antfandakis@yahoo.gr (A.F.); evdo-t@hotmail.com (E.T.); dgiak@auth.gr (D.G.); papadvas@auth.gr (V.N.P.); 2 Laboratory of Histology and Embryology, School of Medicine, Faculty of Health Sciences, Aristotle University of Thessaloniki, 54124 Thessaloniki, Greece; thpapami@auth.gr

**Keywords:** colorectal cancer, preoperative assessment, laboratory testing, staging, carcinoembryonic antigen, preoperative anemia, low skeletal muscle mass, prehabilitation, circulating tumor DNA, mismatch repair, microsatellite instability, Enhanced Recovery After Surgery

## Abstract

Colorectal cancer (CRC) remains one of the most frequently diagnosed malignancies worldwide and a leading indication for major abdominal surgery, with a rising incidence among younger adults. Because CRC resection is a physiologically demanding procedure performed in a frequently comorbid, often anemic and malnourished population, the preoperative period represents a decisive window in which laboratory and diagnostic testing shapes staging, risk stratification, and optimization. This narrative review is deliberately confined to the preoperative window—from histological diagnosis to the day of elective, curative-intent colon or rectal resection—and synthesizes evidence retrieved from PubMed/MEDLINE, Scopus, and ScienceDirect together with current society guidelines. It is organized around four themes: (i) the core laboratory workup, including the complete blood count and preoperative anemia, iron studies, metabolic and hepatic panels, coagulation testing, and carcinoembryonic antigen (CEA); (ii) the diagnostic and staging workup, encompassing complete colonic evaluation, contrast-enhanced computed tomography, pelvic magnetic resonance imaging for rectal cancer, and the selective role of positron emission tomography; (iii) risk stratification and preoperative optimization, spanning patient blood management, nutritional assessment and albumin, computed-tomography-defined low skeletal muscle mass, frailty, functional capacity, prehabilitation, and glycemic control; and (iv) molecular characterization, distinguishing mismatch-repair/microsatellite-instability (MMR/MSI) testing—an established, widely recommended component of CRC evaluation—from genuinely emerging biomarkers such as circulating tumor DNA (ctDNA) and systemic inflammatory and prognostic nutritional indices. Differences between colon and rectal cancer, elective and emergency presentation, and upfront versus post-neoadjuvant surgery are signposted throughout. We further integrate these elements within contemporary guideline and Enhanced Recovery After Surgery (ERAS) frameworks, including the 2025 ERAS Society recommendations for elective colorectal surgery, and distill them into a practical, evidence-based preoperative checklist specifying thresholds, timing, interventions, and strength of supporting evidence. We emphasize that the prognostic power of postoperative ctDNA does not yet translate into ctDNA-guided treatment decisions outside clinical trials. The central message is that preoperative testing in CRC should be purposeful and stage- and risk-adapted rather than reflexive: each investigation should refine staging, modify perioperative management, or enable measurable optimization. Each risk domain is paired with the intervention it should trigger, the criteria identifying candidates for extended thromboprophylaxis are specified, and the systemic inflammatory indices are framed as a trigger for optimization rather than as prognostic commentary.

## 1. Introduction

Colorectal cancer (CRC) is the third most commonly diagnosed malignancy and the second leading cause of cancer-related death worldwide, accounting for approximately 1.9 million new cases and almost one million deaths annually [1]. Global projections indicate that the absolute burden will continue to rise through 2040 as populations grow and age [2,3], while a disproportionate and incompletely explained increase in early-onset disease has extended the at-risk population to younger adults [4]. Surgical resection remains the cornerstone of curative treatment for non-metastatic CRC, and a substantial proportion of these patients ultimately undergo a major colonic or rectal operation.

CRC resection is, however, a physiologically demanding intervention undertaken in a population that is frequently older, comorbid, anemic, and nutritionally depleted. In this context, the preoperative interval is not merely administrative; it is a decisive therapeutic window in which appropriately selected laboratory and diagnostic testing determines accurate staging, quantifies perioperative risk, and identifies modifiable deficits that can be corrected before the physiological insult of surgery. Contemporary practice has moved decisively away from indiscriminate routine panels toward purposeful, indication-driven testing, a principle enshrined in preanesthesia and national testing guidance [5,6]. The guiding question for every investigation is deceptively simple: will the result refine the stage, change perioperative management, or enable measurable optimization? If not, the test rarely adds value.

**Figure 1:**
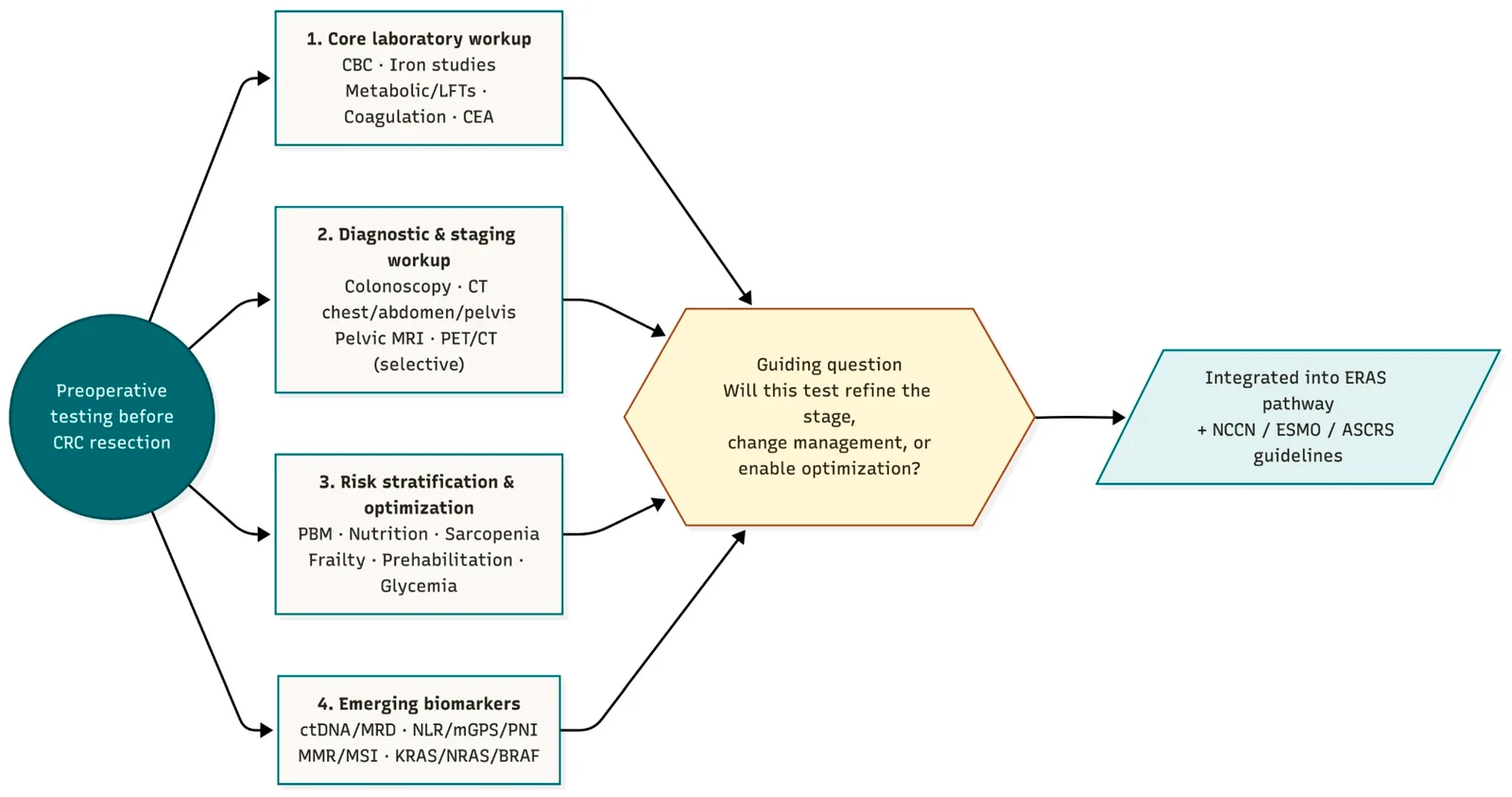
Conceptual framework for preoperative testing before colorectal cancer resection. Four thematic pillars—core laboratory workup, diagnostic and staging workup, risk stratification with optimization, and molecular characterization (established MMR/MSI testing alongside emerging biomarkers)—feed a single decisional filter (whether the test refines staging, changes management, or enables measurable optimization) and converge on an Enhanced Recovery After Surgery (ERAS) pathway aligned with contemporary NCCN, ESMO and ASCRS guidance and the 2025 ERAS Society recommendations. CBC, complete blood count; LFT, liver function test; CEA, carcinoembryonic antigen; PBM, patient blood management; ctDNA, circulating tumor DNA; MRD, molecular residual disease; NLR, neutrophil-to-lymphocyte ratio; mGPS, modified Glasgow Prognostic Score; PNI, prognostic nutritional index; MMR, mismatch repair; MSI, microsatellite instability.

This narrative review addresses what the clinician must remember when ordering and interpreting preoperative tests before CRC resection. We organize the discussion into the core laboratory workup, the diagnostic and staging workup, risk stratification and preoperative optimization, and molecular characterization, and we situate these within current oncological guidelines and Enhanced Recovery After Surgery (ERAS) frameworks. The literature search strategy is detailed in Section 1.1, and the boundaries of the review—together with the distinctions maintained between colon and rectal cancer, elective and emergency presentation, and upfront and post-neoadjuvant surgery—are set out in Section 1.2. The conceptual framework of this review is summarized in Figure 1.

### 1.1. Literature Search Strategy

This article is a narrative review; accordingly, no protocol was registered, and no formal risk-of-bias appraisal or quantitative synthesis was undertaken. The search was nevertheless structured and is reported here so that it can be reproduced. PubMed/MEDLINE, Scopus, and ScienceDirect were searched. Controlled vocabulary and free-text terms describing the population (“colorectal cancer”, “colon cancer”, “rectal cancer”, “colorectal surgery”, “colorectal resection”) were combined using the Boolean operator AND with terms describing the assessment of interest, combined among themselves using OR: “preoperative”, “perioperative”, “preoperative assessment”, “laboratory tests”, “staging”, “computed tomography”, “CT colonography”, “magnetic resonance imaging”, “endorectal ultrasound”, “positron emission tomography”, “carcinoembryonic antigen”, “anemia OR anemia”, “iron deficiency”, “patient blood management”, “albumin”, “malnutrition”, “nutritional screening”, “immunonutrition”, “skeletal muscle”, “sarcopenia”, “body composition”, “frailty”, “cardiopulmonary exercise testing”, “prehabilitation”, “glycemic control”, “venous thromboembolism”, “circulating tumor DNA”, “molecular residual disease”, “microsatellite instability”, “mismatch repair”, and “Enhanced Recovery After Surgery”.

Eligible sources were English-language clinical practice guidelines and consensus statements issued by oncological, colorectal, anesthetic, nutritional, hematological, and enhanced-recovery societies; randomized controlled trials; systematic reviews and meta-analyses; and large prospective, registry, or population-based cohort studies reporting preoperative or peri-diagnostic assessment in adults undergoing resection of colorectal cancer. Publications from the last decade (2016–2025) were prioritized, and earlier work was retained when it remains the primary or landmark evidence for a given practice. Case reports, small single-center series, conference abstracts without full publication, non-English publications, editorials, animal and in vitro studies, and pediatric series were excluded. Records were screened by title and abstract by one author (S.T.); the full texts of potentially eligible records were assessed independently by two authors (S.T. and P.C.), with disagreements resolved by discussion with the senior authors (A.G. and A.G.). Reference lists of the retrieved guidelines and reviews were hand-searched for additional sources, and the websites of NCCN, ESMO, ASCRS, the ERAS Society, ESPEN, ASCO, and NICE were interrogated directly to ensure that the most current version of each recommendation was cited. Where guidance conflicted, the most recently published society document was given precedence and the discrepancy is stated explicitly in the text. Because selection was purposive rather than exhaustive, the possibility of selection bias inherent to the narrative format is acknowledged in Section 8.

### 1.2. Scope, Definitions, and Terminology

The scope of this review is the preoperative interval: the period from histological diagnosis to the day of surgery in adults undergoing elective, curative-intent resection of colon or rectal adenocarcinoma. Topics that are intrinsically postoperative—molecular residual disease detected by ctDNA after resection, selection of adjuvant chemotherapy, oncological surveillance, and extended post-discharge thromboprophylaxis—are considered only to the extent that they justify an action that must be taken before the incision: drawing a baseline CEA and, where a tumor-informed assay is planned, a preoperative plasma sample; securing MMR/MSI status in time for the multidisciplinary discussion of neoadjuvant therapy; and documenting the thrombotic risk profile that determines the duration of prophylaxis prescribed at the preoperative visit. Where the principal utility of a test is postoperative, this is stated, and no preoperative recommendation is derived from it.

Three clinical distinctions are maintained throughout. First, colon and rectal cancer share a common preoperative core—complete colonic evaluation with histological confirmation, baseline CEA, contrast-enhanced CT of the chest, abdomen and pelvis, and widely implemented MMR/MSI testing—but rectal cancer additionally mandates high-resolution pelvic MRI, and its preoperative pathway is far more often reshaped by neoadjuvant treatment. Second, elective surgery permits the full sequence of assessment, optimization, and reassessment described here, whereas emergency presentation with obstruction, perforation, or uncontrolled hemorrhage compresses that window to resuscitation, a focused laboratory panel, contrast-enhanced CT, and—where feasible before any transfusion—a baseline CEA sample; correction of anemia and of nutritional deficits, and prehabilitation, are then deferred to the interval preceding any staged or completion procedure. Third, patients proceeding to upfront resection are staged once, whereas patients operated after neoadjuvant chemoradiotherapy, total neoadjuvant therapy, or immunotherapy require formal restaging before surgery and a repeat baseline of the parameters that treatment itself perturbs, namely hemoglobin, albumin, renal function, glycemia, and skeletal muscle mass. These distinctions are signposted in the relevant subsections, in Section 3.5, and in the checklist presented in Table 1.

To avoid the redundancy that a four-pillar structure invites, one organizing rule is applied throughout. Each investigation is interpreted once and acted upon once: Section 2 and Section 3 establish what a test measures, how to interpret it, and the threshold that defines an abnormal result; Section 4 states what to do about that abnormality and within what timeframe; Section 5 is reserved for molecular characterization of the tumor and for composite indices derived from tests that have already been ordered; and Section 7 collapses the whole into a single checklist. Prognostic evidence is therefore presented once, in the subsection where the test is introduced, and is cross-referenced rather than restated when the corresponding intervention is discussed. Where a parameter necessarily reappears—hemoglobin in Section 2.1 and Section 4.1, albumin in Section 4.2 and Section 5.3, and skeletal muscle mass in Section 4.3 and Section 4.5—the second appearance adds an action and does not repeat the evidence.

## 2. Core Laboratory Workup

### 2.1. Complete Blood Count and Preoperative Anemia

The complete blood count (CBC) is among the most informative baseline laboratory tests in the CRC patient, and preoperative anemia is its most consequential finding. Anemia is defined, following the World Health Organization criteria adopted by perioperative consensus statements, as a hemoglobin concentration below 130 g/L in men and below 120 g/L in women, although a uniform threshold of 130 g/L has been proposed for both sexes in the surgical setting because non-iron-deficient women reach this value [9]. Anemia is present in a large proportion of patients at diagnosis, most commonly reflecting chronic occult blood loss and functional or absolute iron deficiency. Its importance is twofold. First, in a landmark cohort of more than 227,000 non-cardiac surgical patients, even mild preoperative anemia was independently associated with a more than 40% increase in 30-day postoperative mortality and morbidity [8]. Second, in CRC specifically, a meta-analysis of twelve studies encompassing 3588 patients demonstrated that preoperative anemia is associated with significantly worse overall and disease-free survival, with a more pronounced adverse effect in rectal than in colonic disease [7], and subsequent cohorts have reinforced anemia as an independent predictor of impaired long-term survival after resection [23]. The CBC therefore functions simultaneously as a prognostic instrument and as the trigger for a management pathway. The management pathway itself, including the transfusion-related harm that anemia precipitates, is set out once in Section 4.1 and is not anticipated here.

The platelet count and white-cell line complete the picture, informing bleeding risk and flagging occult infection or a myeloproliferative process. A markedly elevated platelet count is not uncommon in CRC and may itself carry prognostic weight when integrated into systemic inflammatory indices (Section 5).

### 2.2. Iron Studies and Iron-Deficiency Anemia

Because iron deficiency is the dominant mechanism of anemia in CRC, a CBC that reveals anemia should prompt iron studies, namely ferritin and transferrin saturation (TSAT), to distinguish absolute from functional deficiency and to guide repletion. Perioperative consensus criteria define absolute iron deficiency as a ferritin below 30 µg/L, whereas in the presence of inflammation (C-reactive protein > 5 mg/L) or a TSAT below 20%, a ferritin below 100 µg/L indicates functional iron deficiency [9]. In practice, this step is frequently omitted: an analysis of electronic medical records found that formal iron-deficiency anemia was documented in only a small minority of CRC patients despite frequent laboratory evidence of deficiency, and only a fraction received iron therapy [10]. Preoperative iron status also carries independent prognostic information in stage II-III disease [11]. The recognition and correction of iron deficiency is thus both a quality-of-care indicator and an actionable optimization target, discussed further in Section 4.1.

### 2.3. Metabolic Panel, Hepatic and Renal Function

A basic metabolic panel establishes baseline electrolytes, renal function, and glucose, while liver function tests screen for the hepatic dysfunction that may accompany metastatic disease. The liver is the most frequent site of CRC metastasis, and although imaging is the definitive staging tool, derangements in transaminases, alkaline phosphatase, and bilirubin may raise suspicion of occult hepatic involvement and inform hepatic reserve when synchronous liver resection is contemplated; composite indices such as the aspartate aminotransferase-to-platelet ratio index predict liver-related morbidity after resection of colorectal liver metastases [12]. Renal function additionally governs the safe administration of iodinated contrast for staging computed tomography and the dosing of renally cleared perioperative drugs and chemotherapy; structured biochemical assessment outperforms questionnaire-based screening for identifying patients at risk of contrast-associated renal injury [24].

### 2.4. Coagulation Testing and Venous Thromboembolism Risk

Routine coagulation screening in unselected patients is neither evidence-based nor recommended; testing should be reserved for patients with liver disease, anticoagulant therapy, or a personal or family history suggestive of a bleeding diathesis [5,6]. The more clinically pressing coagulation issue in CRC is the opposite, namely hypercoagulability. Malignancy and major pelvic–abdominal surgery combine to produce a high venous thromboembolism (VTE) risk; a prospective multicenter cohort reported a postoperative VTE incidence exceeding 11% and derived a validated risk model [13], and large registry data confirm a substantially elevated and durable postoperative thrombotic risk after cancer surgery [25]. These data underpin guideline recommendations for pharmacological prophylaxis begun perioperatively and, for high-risk abdominopelvic cancer surgery, extended for up to four weeks after discharge [14,15,16]. Although the prophylaxis itself is largely delivered after operation, the decision belongs to the preoperative consultation: the risk profile (advanced stage, obesity, prior VTE, thrombophilia, prolonged or open pelvic surgery, reduced mobility) should be documented, the planned duration agreed, and the patient counselled and, where necessary, taught self-injection before admission. Two scoring systems are commonly proposed to formalize that risk profile, and they are not interchangeable. The Caprini risk assessment model, built from 38 weighted clinical variables, stratifies non-orthopedic surgical patients into very low (0–1 points), low (2 points), moderate (3–4 points) and high (5 points or more) risk categories, with a threshold of 9 or more used in several protocols to justify 30 days of prophylaxis [17]. Its discrimination in colorectal cancer surgery specifically is modest: in external validation against three competing models, the Caprini score achieved an area under the curve of 0.661 with a positive predictive value of 0.32, and essentially the entire cohort scored 5 or more, so that strict application would commit every patient to extended prophylaxis; the disease-specific CRC-VTE score performed better (area under the curve 0.728) [18]. The Khorana score, by contrast, was derived and validated in ambulatory patients commencing chemotherapy [19] and should not be transposed to the postoperative setting: in 40,218 patients, its six-month C-statistic was 0.60, fewer than half of all events occurred above the guideline threshold of 2 or more, and the model contains no surgical variable [20]. Our pragmatic recommendation is therefore to use the Caprini score as a structured prompt rather than as a decision rule, to record it alongside the disease- and operation-specific features that carry the greatest weight (advanced stage, open or prolonged pelvic surgery, age above 70 years, previous venous thromboembolism or known thrombophilia, obesity, reduced mobility, and a raised preoperative D-dimer), and to reserve extended prophylaxis for patients scoring 5 or more who also carry at least one of those features, and for essentially patients scoring 9 or more. The evidence supporting the duration is consistent: in a meta-analysis of 2936 patients undergoing colorectal surgery, extended prophylaxis for at least 28 days reduced 30-day venous thromboembolism from 3.6% to 0.71% (odds ratio 0.20, 95% confidence interval 0.10–0.42) without a significant excess of major bleeding [26].

### 2.5. Carcinoembryonic Antigen (CEA)

Preoperative serum CEA is the one tumor marker with a firmly established role in CRC. Guidelines from ASCO, ESMO, NCCN, and ASCRS recommend its measurement at diagnosis in patients in whom the result would inform management, both to establish a baseline for postoperative surveillance and to contribute prognostic information [27,28,29,30]. An elevated preoperative CEA (commonly dichotomized at >5 ng/mL) is independently associated with worse recurrence-free and overall survival across stages, a relationship that holds even after adjustment for stage [21,22]. A normal preoperative value does not exclude disease, and a persistently or newly elevated postoperative CEA remains the most sensitive biochemical trigger for recurrence during follow-up [31]. The preoperative action is therefore narrow but obligatory: obtain the baseline value before resection, because a postoperative CEA can only be interpreted against it.

## 3. Diagnostic and Staging Workup

### 3.1. Complete Colonic Evaluation

**Figure 2:**
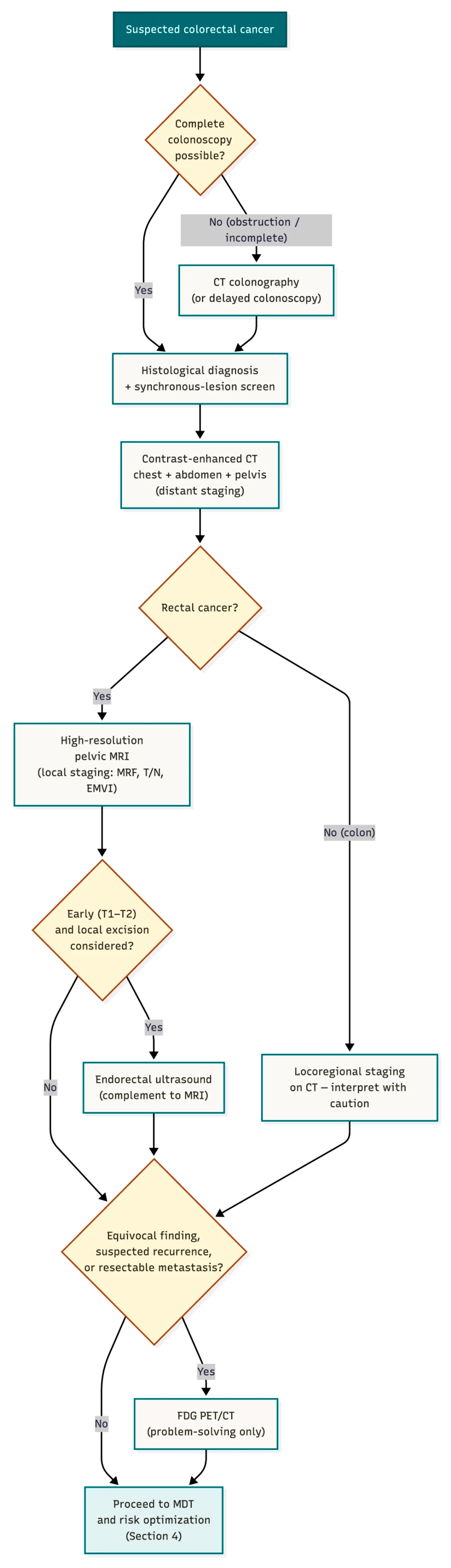
Preoperative diagnostic and staging algorithm for colorectal cancer. Complete colonic evaluation and contrast-enhanced computed tomography form the strongly recommended backbone for patients; high-resolution pelvic magnetic resonance imaging is added for every rectal tumor, with endorectal ultrasound reserved for early cancers considered for local excision, and fluorodeoxyglucose positron emission tomography/computed tomography restricted to problem-solving. The algorithm assumes an elective presentation; the abbreviated emergency pathway and the restaging pathway after neoadjuvant therapy are described in Section 3.5. MRF, mesorectal fascia; EMVI, extramural vascular invasion; MDT, multidisciplinary team.

The preoperative diagnostic and staging algorithm applied throughout this section is summarized in Figure 2. Complete evaluation of the entire colon before resection is strongly recommended, both to confirm the diagnosis histologically and to detect synchronous neoplasia, which is present in a clinically meaningful minority of patients. A contemporary colonoscopy series found that nearly half of CRC cases harbored synchronous polyps, underscoring the consequences of an incomplete examination for operative planning [32]. When colonoscopy cannot reach the caecum, most often because of an obstructing left-sided tumor, computed tomographic (CT) colonography is the preferred completion strategy, reliably localizing the index tumor and detecting proximal synchronous lesions, and altering the planned operation in a non-trivial proportion of patients [33,34]. Where preoperative completion is impossible, evaluation of the residual colon should be scheduled early after resection.

### 3.2. Contrast-Enhanced Computed Tomography

Contrast-enhanced CT of the chest, abdomen, and pelvis is the standard modality for distant staging in both colon and rectal cancer, screening for hepatic and pulmonary metastases and for peritoneal or nodal disease [27,28]. Its accuracy for locoregional T- and N-staging of colon cancer is, however, only moderate: a nationwide study reported limited concordance between CT-based staging and definitive pathology [35], and a systematic review confirmed the constrained sensitivity of cross-sectional imaging for nodal status [36]. CT therefore excels at answering the question that most often changes the operative plan, the presence of distant disease, while its locoregional assessment should be interpreted with appropriate caution.

### 3.3. Pelvic Magnetic Resonance Imaging for Rectal Cancer

For rectal cancer, high-resolution pelvic magnetic resonance imaging (MRI) is the reference standard for local staging and is mandated by all major guidelines [37,38,39,40]. MRI defines the relationship of the tumor to the mesorectal fascia, T- and N-category, and extramural vascular invasion, and these features drive the selection of neoadjuvant therapy and the surgical approach. Endorectal ultrasound remains a valuable complementary tool, with meta-analytic data indicating diagnostic accuracy comparable to MRI for T-staging, and a particular advantage for early (T1–T2) tumors being considered for local excision [41]. The combination of accurate local staging by MRI and distant staging by CT constitutes the imaging backbone of modern rectal cancer management.

### 3.4. Positron Emission Tomography and Problem-Solving Imaging

Fluorine-18 fluorodeoxyglucose positron emission tomography combined with CT (FDG PET/CT) is not recommended for routine initial staging [29]. Its value is selective: in the evaluation of equivocal findings, suspected recurrence, or potentially resectable metastatic disease, PET/CT can detect occult lesions and alter management in a defined subset of patients [42,43]. Judicious, question-driven use, rather than reflexive incorporation into the primary staging pathway, characterizes appropriate practice.

### 3.5. Elective Versus Emergency Presentation and Restaging After Neoadjuvant Therapy

The staging sequence described above assumes an elective presentation. In obstruction, perforation, or uncontrolled hemorrhage, the preoperative workup is necessarily abbreviated: contrast-enhanced CT of the chest, abdomen and pelvis remains achievable and should be obtained because it still answers the question that most often alters the operation, namely the presence of distant disease; a focused laboratory panel (CBC, electrolytes, renal function, lactate, coagulation, group and save) replaces the full elective panel; and complete colonic evaluation, iron repletion, nutritional optimization, and prehabilitation are deferred to the interval before any staged or completion resection. A baseline CEA should still be drawn where the sample can be taken before transfusion, and the resected specimen must be submitted for MMR/MSI testing even when the operation was unplanned, because the Lynch-syndrome and immunotherapy implications are unchanged.

Patients treated with neoadjuvant chemoradiotherapy, total neoadjuvant therapy, or—in deficient-MMR tumors—neoadjuvant immunotherapy require a second, pre-surgical assessment rather than a repetition of the initial one. Restaging pelvic MRI, interpreted with awareness of treatment-related fibrosis, defines the residual tumor–mesorectal fascia relationship and informs the decision between resection and organ preservation, while repeat CT of the chest, abdomen and pelvis excludes interval distant progression [39,40]. Equally important is the recognition that the interval treatment itself perturbs the very parameters measured at diagnosis: hemoglobin, albumin, renal function, glycemia and skeletal muscle mass frequently deteriorate during chemoradiotherapy, so the laboratory and body-composition baseline must be re-established close to the planned operation rather than carried forward from the pre-treatment visit. In this population, the optimization interventions described in Section 4 are best delivered during, rather than after, neoadjuvant therapy, when several weeks of usable time remain (Table 2).

## 4. Risk Stratification and Preoperative Optimization

**Figure 3:**
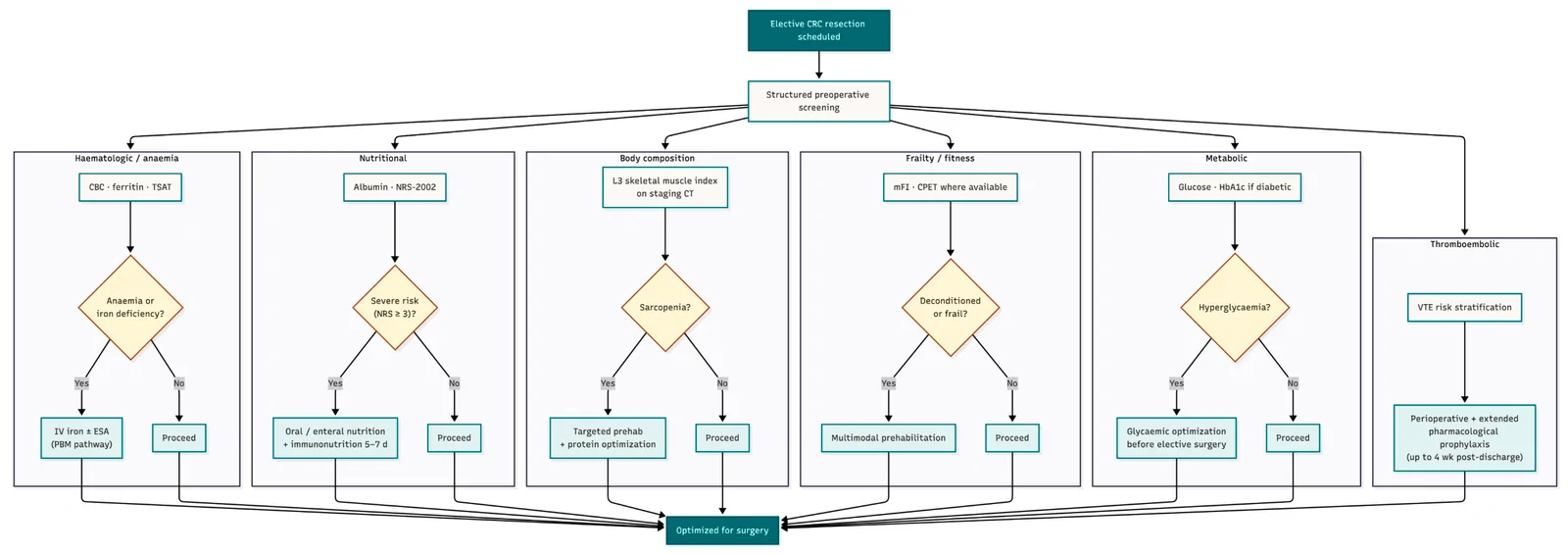
Preoperative optimization pathway across six modifiable risk domains—hematologic, nutritional, body composition, frailty and fitness, metabolic, and thromboembolic. Each domain follows the same three steps: standardized assessment, decision, and evidence-based intervention where a deficit is identified. CBC, complete blood count; TSAT, transferrin saturation; ESA, erythropoiesis-stimulating agent; PBM, patient blood management; NRS-2002, Nutritional Risk Screening 2002; mFI, modified frailty index; CPET, cardiopulmonary exercise testing; VTE, venous thromboembolism.

Beyond diagnosis and staging, the preoperative period offers the opportunity to measure and modify risk. Figure 3 summarizes the multidomain optimization pathway detailed in the subsections that follow. Several deficits identifiable by simple tests, namely anemia, hypoalbuminemia, CT-defined low skeletal muscle mass, frailty, poor fitness, and hyperglycemia, are both prognostic and, to varying degrees, correctable.

### 4.1. Preoperative Anemia Management and Patient Blood Management

Anemia detected as described in Section 2.1 is the most modifiable of the preoperative deficits, and its correction is the central optimization target of patient blood management (PBM) frameworks; the prognostic evidence is not repeated here. Two harms are averted: the independent effect of anemia itself, and the infectious and oncological risk attached to the perioperative allogeneic transfusion that anemia precipitates. International consensus statements recommend the early detection and treatment of anemia and iron deficiency before major elective surgery, with a preoperative hemoglobin target and a preference for intravenous iron when the interval to surgery is short [9,44]. In CRC specifically, the randomized IVICA and FIT trials compared intravenous with oral iron: both demonstrated that intravenous iron more effectively and rapidly replenishes iron stores and improves hemoglobin and quality of life, although neither established a reduction in transfusion as a certain benefit and preoperative hemoglobin normalization remained difficult to achieve within typical surgical timelines [45,46]. Observational data likewise show meaningful preoperative hemoglobin gains with intravenous iron [47]. The pragmatic message is to screen early, investigate iron status, and treat deficiency in time for it to matter (Table 3).

### 4.2. Nutritional Assessment, Albumin, and Immunonutrition

Malnutrition is common in CRC and strongly predicts adverse outcomes. Serum albumin, although an imperfect and inflammation-sensitive marker, remains one of the most powerful single preoperative predictors of postoperative mortality and morbidity, as demonstrated across more than 54,000 operations in the National VA Surgical Risk Study [48], with the association confirmed in colorectal-specific analyses [49]. Structured nutritional screening identifies patients at risk and predicts complications [50], and ESPEN guidance recommends correcting severe nutritional risk before major cancer surgery, delaying non-urgent operations where necessary [51]. In practical terms, a Nutritional Risk Screening 2002 score of 3 or more, unintentional weight loss exceeding 10% (or 5% within three months), a body mass index below 18.5 kg/m^2^, or an albumin below 30 g/L identifies the patient in whom oral nutritional supplementation, ideally for 7–14 days before operation, is indicated [51]. Perioperative immunonutrition reduces infectious complications and length of stay in gastrointestinal cancer surgery in meta-analytic data [52]. In colorectal cancer specifically, meta-analyses likewise show that perioperative and preoperative immunonutrition reduces infectious complications [53,54]; the 2025 ERAS Society recommendations restrict immunonutrition to patients identified as malnourished rather than endorsing it for all comers [55]. Nutritional optimization is therefore among the most actionable of preoperative interventions. Albumin recurs in Section 5.3 only as a component of composite inflammatory–nutritional indices; the evidence for albumin as a single preoperative marker is given here and is not restated there (Table 3).

### 4.3. CT-Defined Low Skeletal Muscle Mass

Sarcopenia, as currently defined by the European Working Group on Sarcopenia in Older People (EWGSOP2), is a skeletal muscle disease in which low muscle strength is the primary criterion, low muscle quantity or quality is confirmatory, and poor physical performance denotes severity [56]. Almost none of the surgical oncology literature applies this composite definition. What is measured instead, and what is reported below, is the cross-sectional skeletal muscle area at the level of the third lumbar vertebra on the staging CT already obtained, normalized to the square of height to yield the skeletal muscle index (SMI). Throughout this review, we therefore use the term CT-defined low skeletal muscle mass, and reserve “sarcopenia” for the studies that use that word, because the two are not interchangeable: a patient may have a low SMI with preserved strength, and vice versa. This distinction matters because the reported cutoffs are neither widely implemented nor interchangeable. The most widely applied thresholds are those of Prado et al. (SMI < 52.4 cm^2^/m^2^ in men and <38.5 cm^2^/m^2^ in women) [57] and the BMI-stratified thresholds of Martin et al. [58], while Asian cohorts apply substantially lower values; the software used for segmentation, the choice of Hounsfield-unit range, and whether muscle radiodensity is co-reported all vary further between studies. Prevalence estimates and effect sizes must be read with this heterogeneity in mind. With that caveat stated, the prognostic signal is consistent. Meta-analyses demonstrate that CRC patients with CT-defined low skeletal muscle mass experience significantly more postoperative complications and worse overall, disease-free, and cancer-specific survival [59,60], and a large colon cancer cohort confirmed that low muscle mass and low muscle radiodensity independently predict short-term mortality and long-term survival [61]. In rectal cancer specifically, meta-analyses report the same association with postoperative complications and long-term survival [62,63]. Because the measurement exploits imaging that is performed regardless, it represents a high-value, essentially cost-free addition to preoperative risk stratification; what it does not yet provide is a validated, internationally agreed threshold at which a specific intervention is triggered.

In a secondary analysis of the PREHAB randomized trial, four weeks of multimodal prehabilitation improved nutritional status and increased muscle strength by 27% relative to standard care [64]; and in rectal cancer patients undergoing neoadjuvant treatment, a multimodal program produced a net gain in muscle mass where the comparison cohort lost muscle, the gain being associated with earlier return of stoma function, earlier tolerance of a soft diet, and fewer surgical-site infections [65] (Table 3).

### 4.4. Frailty and Functional Capacity

Frailty, distinct from chronological age, predicts adverse surgical outcomes. The modified frailty index, derived from routinely available clinical variables, is associated with increased morbidity, mortality, prolonged stay, and non-home discharge in colorectal and gastrointestinal cancer surgery [66,67], and a systematic review and meta-analysis confirms that frailty independently predicts worse oncological outcomes in colorectal cancer surgery [68]. Objective functional capacity likewise predicts outcome: the international METS study demonstrated that subjective clinical assessment of fitness is unreliable, whereas objective measures such as cardiopulmonary exercise testing (CPET) and biomarkers perform better [69], and CPET-derived variables discriminate postoperative morbidity risk after rectal cancer surgery [70]. Where resources permit, objective fitness assessment refines the risk estimate and identifies candidates for prehabilitation (Table 3).

### 4.5. Prehabilitation

Prehabilitation, the multimodal enhancement of physiological reserve before surgery through exercise, nutritional support, and psychological preparation, operationalizes the optimization philosophy. An early randomized trial showed superior recovery of functional walking capacity with prehabilitation compared with postoperative rehabilitation [71], and the multicenter PREHAB randomized trial subsequently demonstrated a reduction in severe and medical postoperative complications and improved functional recovery after CRC surgery [72]. A Cochrane review found that prehabilitation probably improves preoperative fitness, while cautioning that the certainty of evidence for complication and quality-of-life endpoints remains limited by heterogeneity [73]. Reflecting precisely this heterogeneity of program content, intensity, and endpoint definition, the 2025 ERAS Society recommendations for elective colorectal surgery no longer make a specific recommendation in favor of prehabilitation, in contrast to the qualified endorsement of the 2018 document [55,74]. The reasonable current position is therefore selective rather than widely implemented: prehabilitation should be offered preferentially to the deconditioned, frail, or nutritionally depleted patient in whom several weeks are available before operation, and ideally within a service that audits its own outcomes (Table 3).

### 4.6. Glycemic Control

Perioperative hyperglycemia, whether or not the patient carries a diabetes diagnosis, is associated with a roughly twofold increase in surgical site infection, reoperation, and death, and treatment of day-of-surgery hyperglycemia appears to attenuate this risk [75]. Preoperative assessment of glycemic status, namely glucose and, in known or suspected diabetes, glycated hemoglobin (HbA1c), identifies patients who warrant optimization before elective resection. An HbA1c of 8.5% (69 mmol/mol) or above generally warrants referral for medical optimization before a non-urgent resection, an HbA1c of 6.5% (48 mmol/mol) or above in a patient not known to be diabetic establishes the diagnosis and should prompt referral, and a day-of-surgery glucose above 10 mmol/L (180 mg/dL) should be actively treated rather than observed [6,75] (Table 3).

### 4.7. Composite Risk Prediction

Several instruments integrate these variables into quantitative risk estimates. The colorectal-specific CR-POSSUM model predicts operative mortality more accurately than generic scores in colorectal populations [76], although its performance varies with case-mix and benefits from local recalibration. The ACS-NSQIP surgical risk calculator provides individualized estimates but shows imperfect calibration in elderly CRC cohorts [77], and the Revised Cardiac Risk Index remains a standard tool for the cardiac component of risk [78]. Such calculators inform, but do not replace, holistic clinical judgment and shared decision-making. Two points prevent this subsection from duplicating those above. First, the composite calculators take the individual domains of Section 4.1, Section 4.2, Section 4.3, Section 4.4, Section 4.5 and Section 4.6 as inputs and return a single probability; they are not a second, independent assessment of the same deficits, which is why the domain-specific thresholds appear only once, in the subsection where each domain is introduced. Second, a composite score and a domain-specific deficit answer different questions: the calculator informs consent and the choice between operative and nonoperative management, whereas the domain-specific abnormality identifies something that can actually be corrected before the incision (Table 3).

**Table 3:** Preoperative risk domains and optimization targets in colorectal cancer resection. PBM, patient blood management; CPET, cardiopulmonary exercise testing; SSI, surgical site infection; NRS-2002, Nutritional Risk Screening 2002; TSAT, transferrin saturation; BMI, body mass index; HbA1c, glycated hemoglobin; EWGSOP2, European Working Group on Sarcopenia in Older People 2.

Domain	Preoperative Assessment	Optimization Target/Evidence
Anemia/iron	Hemoglobin, ferritin, TSAT	Early IV iron; PBM pathways [44,45].
Nutrition	Albumin, screening tools (e.g., NRS-2002)	Correct severe risk; immunonutrition in malnourished patients [51,52,55]. Trigger: NRS-2002 ≥ 3, weight loss > 10%, BMI < 18.5 kg/m^2^, or albumin < 30 g/L.
CT-defined low skeletal muscle mass	L3 skeletal muscle index on staging CT (Prado or Martin cutoffs; not equivalent to EWGSOP2 sarcopenia)	Prognostic; supports targeted prehabilitation [59,61]. No agreed intervention threshold [56,57,58]. Action: protein 1.2–1.5 g/kg/day plus supervised resistance exercise for 4 weeks or more; conditional recommendation, low-to-moderate certainty [64,65].
Frailty/fitness	mFI; CPET where available	Objective fitness outperforms subjective [66,69].
Prehabilitation	Baseline fitness, nutrition, psychology	Reduces complications in an RCT [72,73]; no specific recommendation in ERAS 2025-offer selectively to the deconditioned [55].
Glycemic control	Glucose; HbA1c if diabetic or suspected	Hyperglycemia doubles SSI risk [75]. Optimize if HbA1c ≥ 8.5% (69 mmol/mol); treat day-of-surgery glucose > 10 mmol/L.
Systemic inflammation	mGPS from CRP and albumin; NLR and PNI from the CBC (no extra tests)	Prognostic complement to TNM stage; mGPS 1–2 or PNI < 45 triggers escalation to structured optimization, not a change of operation [79,80].
Thromboembolic risk	Caprini score; disease- and operation-specific features; preoperative D-dimer	Extended LMWH for 28 days or more if Caprini 5 or more plus a high-risk feature, or Caprini 9 or more; reduces 30-day VTE from 3.6% to 0.71% [17,18,26].

## 5. Established Molecular Testing and Emerging Biomarkers

**Figure 4:**
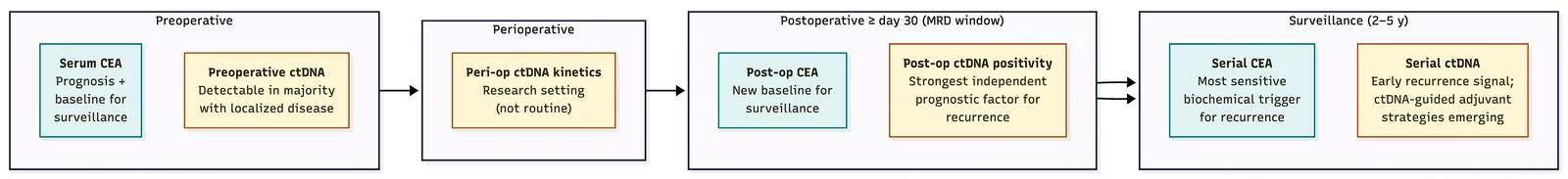
Longitudinal role of carcinoembryonic antigen (CEA, teal) and circulating tumor DNA (ctDNA, amber) across the preoperative, perioperative, postoperative and surveillance phases of colorectal cancer resection. CEA remains the established baseline and recurrence-monitoring biomarker; postoperative ctDNA is a powerful independent prognostic indicator of molecular residual disease (MRD), but current guidelines regard the evidence as insufficient to recommend ctDNA-guided treatment decisions outside clinical trials. The preoperative actions shown are the acquisition of a baseline CEA in every patient and, only where a tumor-informed assay is planned within a trial or research protocol, the collection of tumor tissue and a pre-resection plasma sample.

Molecular and biomarker testing is presented last not because it is peripheral but because it operates on a different axis from everything above: it characterizes the tumor and the host response rather than the physiological fitness of the patient. Two clarifications keep this section connected to the rest of the review. First, the assays discussed here span the full evidential spectrum and are deliberately ordered along it: MMR/MSI testing is established and mandated for every patient, and is standard of care rather than an emerging marker; the systemic inflammatory and nutritional indices are derived at no additional cost from blood already drawn in Section 2 and are prognostic adjuncts; and ctDNA remains investigational as a determinant of treatment. Second, each has a defined relationship to material already covered—MMR/MSI status determines whether the neoadjuvant pathway of Section 3.5 is available at all; the inflammatory indices are computed from the complete blood count of Section 2.1 and the albumin of Section 4.2 and are interpreted alongside the frailty assessment of Section 4.4; and ctDNA is the longitudinal counterpart of the baseline CEA of Section 2.5. Table 4 and Figure 4 present these relationships together.

### 5.1. Mismatch-Repair and Microsatellite-Instability Testing: An Established Requirement, Not an Emerging Biomarker

Determination of mismatch-repair (MMR) status by immunohistochemistry or of microsatellite instability (MSI) by molecular testing is not an emerging biomarker but an established, widely recommended component of colorectal cancer evaluation, mandated for every newly diagnosed tumor by NCCN, ESMO, ASCRS, and the joint ASCP/CAP/AMP/ASCO molecular-biomarker guideline [27,29,37,81,82]. It is included in this section for completeness of molecular characterization, and its evidence maturity in Table 4 is graded accordingly. Ideally it is performed on the diagnostic biopsy, so that the result is available for the multidisciplinary discussion that precedes any decision about neoadjuvant therapy, rather than on the resection specimen. Preoperatively and peri-diagnostically, MMR/MSI status carries prognostic weight, with deficient MMR (MSI-high) conferring a favorable prognosis in stage II-III disease, and predictive weight, identifying tumors that respond poorly to fluoropyrimidine monotherapy yet are exquisitely sensitive to immune checkpoint inhibition [95]. Two consequences make the timing of this test a genuinely preoperative issue. First, a deficient-MMR result may change whether, and in what form, surgery is performed at all: in locally advanced deficient-MMR rectal cancer, six months of neoadjuvant dostarlimab produced clinical complete responses that permitted nonoperative management without chemoradiotherapy or resection [83], while in deficient-MMR colon cancer, the NICHE-2 trial reported major pathological response in 98% and pathological complete response in 68% of patients after short-course neoadjuvant nivolumab and ipilimumab [84]. A patient whose deficient-MMR status is discovered only on the resection specimen has lost access to these pathways. Second, deficient MMR triggers formal evaluation for Lynch syndrome—germline testing of MLH1, MSH2, MSH6, PMS2 and EPCAM, with MLH1 promoter hypermethylation and BRAF V600E testing used to identify sporadic hypermethylated tumors—which carries implications for the extent of resection (a discussion of extended or total colectomy in a young patient with confirmed Lynch syndrome), for postoperative surveillance, and for cascade screening of first-degree relatives. Genetic counseling is therefore best initiated before, not after, the operation. Somatic mutations in KRAS, NRAS, and BRAF, while principally relevant to systemic therapy in advanced disease, additionally carry prognostic information, with BRAF and, in microsatellite-stable tumors, KRAS mutation associated with worse survival [93,94]. Knowledge of molecular status increasingly informs the multidisciplinary plan into which surgery is embedded. To state the position without ambiguity, MMR/MSI testing is standard of care for every patient with colorectal cancer, belongs to the strongly recommended diagnostic workup rather than to any list of emerging biomarkers, and rests on two justifications that are already actionable at the time of diagnosis. It guides systemic therapy, by identifying the tumors that derive little benefit from fluoropyrimidine monotherapy and pronounced benefit from checkpoint inhibition, and it initiates screening for Lynch syndrome in the patient and, through cascade testing, in the family. A colorectal cancer resection performed without a documented MMR/MSI result should be regarded as a deviation from the standard of care rather than as an acceptable omission, and the result should be recorded in the operative planning documentation alongside the stage.

### 5.2. Circulating Tumor DNA and Molecular Residual Disease: Prognostic, Not Yet Decision-Guiding

Circulating tumor DNA (ctDNA) is the most intensively studied emerging biomarker in colorectal cancer, but its established value is prognostic and predominantly postoperative, and the preoperative clinician should be clear about that distinction. Preoperative ctDNA is detectable in the majority of patients with localized disease; however, its detection rate falls with lower stage and it neither refines TNM staging nor alters the operative plan, so preoperative ctDNA has no current role outside research protocols. What is powerful is the postoperative signal: persistence of ctDNA after resection, a surrogate for molecular residual disease (MRD), is among the strongest independent predictors of recurrence [96]. In the DYNAMIC randomized trial, a ctDNA-guided strategy reduced adjuvant chemotherapy use without compromising two-year recurrence-free survival in stage II colon cancer [85], and the GALAXY arm of CIRCULATE-Japan established postoperative ctDNA positivity as the strongest independent prognostic factor and a predictor of adjuvant chemotherapy benefit [86]; a meta-analysis confirmed a markedly elevated recurrence risk with ctDNA positivity, more pronounced with tumor-informed assays [97]. That evidence has not, however, matured into a mandate. In the randomized phase 2/3 DYNAMIC-III trial in stage III colon cancer, ctDNA again separated prognostic groups decisively (three-year recurrence-free survival 87% in ctDNA-negative versus 49% in ctDNA-positive patients), yet ctDNA-guided de-escalation did not meet its non-inferiority margin against standard adjuvant therapy (85.3% versus 88.1%), and treatment escalation in ctDNA-positive patients conferred no benefit [87]. Accordingly, the NCCN guidelines state that ctDNA is prognostic but not predictive in the adjuvant treatment of microsatellite-stable/MMR-proficient disease, that there is insufficient evidence to recommend routine use of ctDNA assays outside clinical trials, and that de-escalation and treatment decision-making should not be based on ctDNA findings; ASCO likewise advises that, outside a trial, ctDNA testing be offered only where the result could genuinely inform a clinical decision [27,37,88]. Assay standardization, tumor-informed versus tumor-agnostic design, turnaround time, and cost remain unresolved. The preoperative implication is correspondingly narrow but real: where a tumor-informed assay is planned, tissue and a pre-resection plasma sample must be secured before operation, and the patient should be counselled that the result is prognostic information gathered within, or in preparation for, a clinical trial rather than a determinant of therapy. Figure 4 illustrates the complementary longitudinal roles of CEA and ctDNA across the preoperative, perioperative, postoperative and surveillance phases. CEA is the biomarker that must be measured before surgery and is interpretable in every setting, whereas ctDNA is the biomarker whose value accrues after surgery and, for the present, within trials. Step 3 of the checklist in Table 5 accordingly mandates a baseline CEA, and no step mandates ctDNA.

### 5.3. Systemic Inflammatory and Nutritional Indices

Inexpensive indices derived from the CBC and albumin capture the host inflammatory and nutritional response and carry consistent prognostic value. An elevated preoperative neutrophil-to-lymphocyte ratio (NLR) is associated with worse survival in meta-analyses [89,90], and the modified Glasgow Prognostic Score (mGPS), which combines C-reactive protein and albumin, has been validated across thousands of patients [91]; composite scores integrating mGPS and NLR further refine stratification [100]. The Onodera Prognostic Nutritional Index, calculated from albumin and lymphocyte count, similarly predicts survival [92]. Because these indices require no additional testing beyond routine bloods, they represent efficient adjuncts to conventional staging.

Three recommendations follow from the evidence, together with one caution. First, they should be computed rather than ordered: NLR, PLR, mGPS and PNI all derive from the complete blood count, albumin and C-reactive protein already obtained in Section 2, so the marginal cost of reporting them is zero and there is no justification for additional phlebotomy. Second, they are best used as a host-staging complement to tumor staging rather than as a stand-alone prognostic statement: in a large cohort undergoing potentially curative resection, combining TNM stage with mGPS separated five-year cancer-specific survival from 97% in stage I with mGPS 0 to 32% in stage III with mGPS 2, a spread that neither variable achieves alone [79]. Third, and most practically, an elevated mGPS or a low PNI should lower the threshold for the interventions already described rather than generate a parallel pathway. Systemic inflammation, malnutrition, low skeletal muscle mass and frailty co-segregate: in 1002 patients undergoing colorectal cancer resection, an mFI-5 score of 2 or more and an elevated systemic inflammatory grade were each independently associated with postoperative complications [80]. A patient with an adverse index should therefore be referred for formal nutritional assessment (Section 4.2), have body composition read from the staging CT (Section 4.3), undergo objective frailty and fitness assessment (Section 4.4), and be prioritized for prehabilitation (Section 4.5). In our practice, an mGPS of 1 or 2, or a PNI below 45, is treated as sufficient reason to escalate a patient from routine to structured preoperative optimization and to seek an anesthetic opinion earlier than the operative date would otherwise dictate. The caution matters equally: these indices are prognostic rather than predictive, no treatment threshold has been validated against an outcome, and they must not be used to withhold or delay a curative resection, to select the operative approach, or to substitute for formal anesthetic and oncological assessment. Their legitimate uses are triage to optimization, informed consent, and multidisciplinary discussion.

## 6. Integration Within Guidelines and Enhanced Recovery Pathways

**Figure 5:**
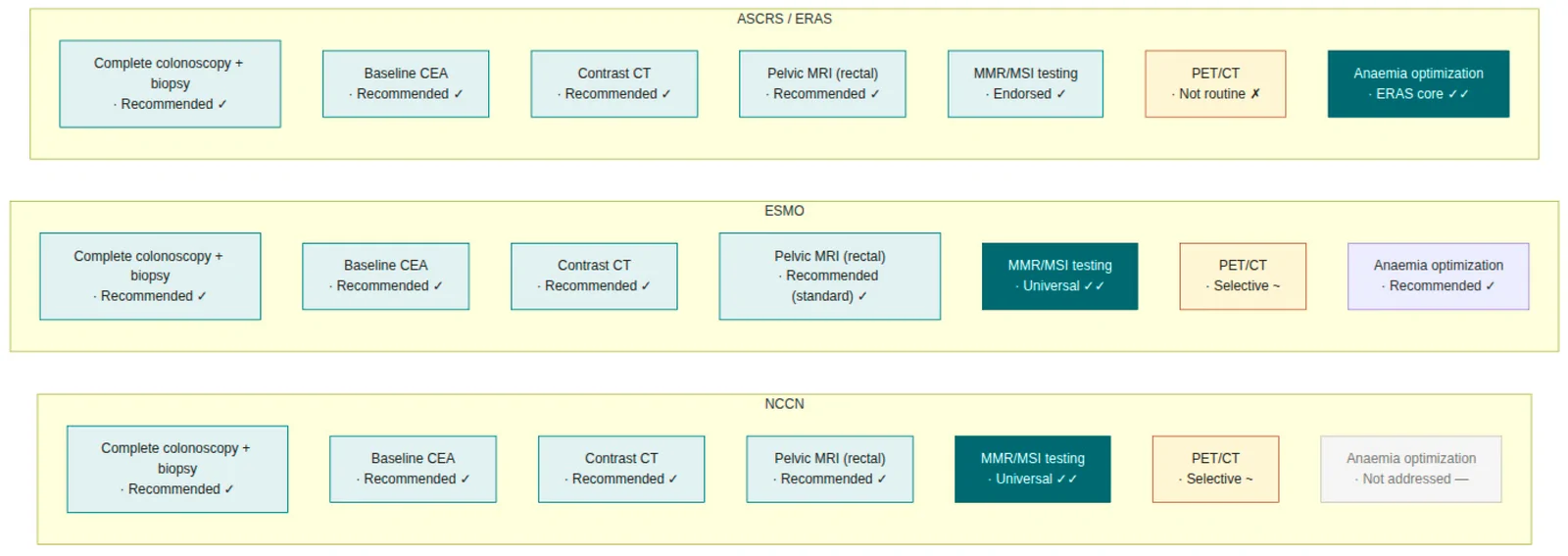
Graphical convergence of the National Comprehensive Cancer Network (NCCN), European Society for Medical Oncology (ESMO) and American Society of Colon and Rectal Surgeons/Enhanced Recovery After Surgery (ASCRS/ERAS) guidelines on the preoperative workup for colorectal cancer. Green shading denotes explicit recommendation, teal denotes widely implemented or core status, amber denotes selective indication, and grey denotes elements not addressed by the guideline. Readers should consult the current version of each guideline for exact wording and evidence grading.

The individual tests discussed above acquire coherence only when integrated into a structured pathway. The major oncological guidelines, namely NCCN, ESMO, and ASCRS, converge on a common preoperative core for CRC: histological confirmation with complete colonic evaluation, baseline CEA, contrast CT of the chest, abdomen, and pelvis for distant staging, pelvic MRI for rectal cancer, and widely implemented MMR/MSI testing [27,28,29,37,38,39,40]. They differ mainly in emphasis and in the selective role assigned to adjuncts such as PET/CT and endorectal ultrasound, and in their explicit position on ctDNA, which all three currently place outside routine practice (Figure 5) (Table 6).

The ERAS Society guidelines for elective colorectal surgery embed preoperative testing within a broader program designed to attenuate the surgical stress response. These recommendations were updated in 2025, superseding the 2018 and 2013 documents cited in earlier reviews [74,101]; the 2025 version applies a new and more rigorous evidence-appraisal methodology and, where it differs from its predecessor, is the version followed here [55]. Its preoperative components remain structured preadmission education and counseling, formal risk and comorbidity assessment, nutritional screening, correction of anemia, avoidance of prolonged fasting, and preoperative carbohydrate loading, translating the evidence of Section 2, Section 3 and Section 4 into a reproducible workflow. Three changes are relevant to the present review: prehabilitation is no longer the subject of a specific recommendation, reflecting persistent heterogeneity in program design and outcome reporting; immunonutrition is now positioned for patients identified as malnourished rather than for all patients; and a slightly positive intraoperative fluid balance is preferred [55]. Enhanced recovery pathways as a whole reduce complications and length of stay without increasing readmission in colorectal surgery, as shown in a Cochrane review and multiple meta-analyses [98,99]. Preoperative testing is thus not an isolated checklist but the front end of an integrated perioperative system.

## 7. A Practical Preoperative Checklist

**Figure 6:**
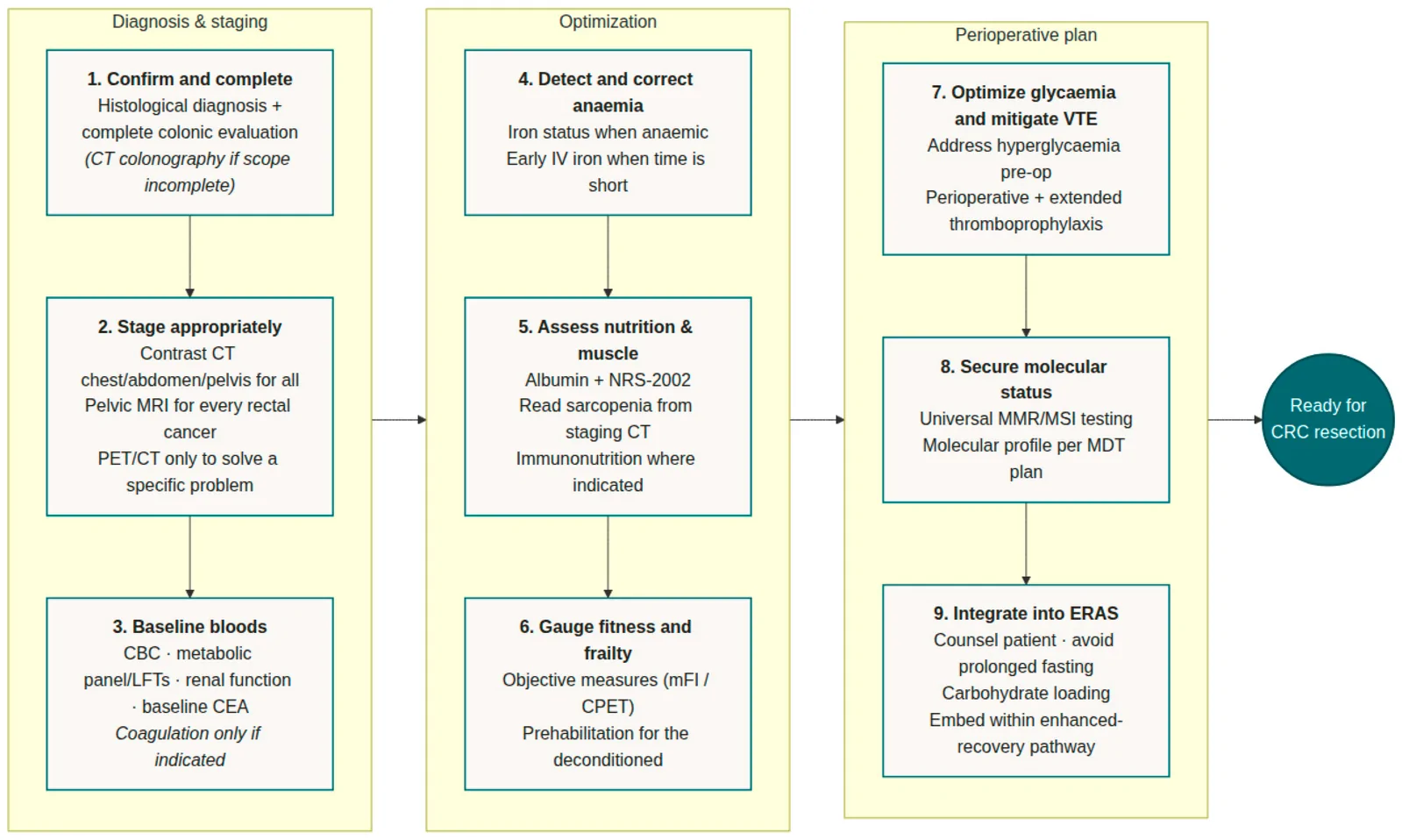
Nine-step practical preoperative checklist for colorectal cancer resection. Each step is purposeful and evidence-anchored; the sequence integrates diagnostic confirmation, staging, laboratory baseline, correction of anemia and nutrition, functional optimization, thromboprophylaxis and molecular characterization into a single Enhanced Recovery After Surgery (ERAS)-compatible workflow. CBC, complete blood count; LFT, liver function test; CEA, carcinoembryonic antigen; NRS-2002, Nutritional Risk Screening 2002; mFI, modified frailty index; CPET, cardiopulmonary exercise testing; VTE, venous thromboembolism; MMR, mismatch repair; MSI, microsatellite instability; MDT, multidisciplinary team; ERAS, Enhanced Recovery After Surgery.

Synthesizing the evidence, the clinician preparing a patient for CRC resection must remember a compact set of purposeful actions. Table 5 states, for each step, the threshold or trigger that defines an abnormal result, the intervention indicated, the timing in which it must be delivered to be useful, and the strength of the supporting evidence, so that the checklist can be applied rather than merely read. It is offered as an aide-memoire rather than a rigid protocol, to be adapted to local resources and the individual patient; a graphical version is provided in Figure 6.

## 8. Limitations and Future Directions

This review is narrative rather than systematic, and the evidence it summarizes is heterogeneous: much of the prognostic literature on anemia, CT-defined low skeletal muscle mass, and inflammatory indices is observational and susceptible to confounding, while the certainty of evidence for prehabilitation endpoints beyond fitness remains limited [73]. Although the search described in Section 1.1 was structured, it was purposive rather than exhaustive: no protocol was registered, screening of titles and abstracts was performed by a single author, no formal risk-of-bias or GRADE appraisal was undertaken, and the restriction to English-language publications may have excluded relevant evidence, particularly the Asian body-composition literature in which different skeletal-muscle-index cutoffs apply. Grey literature and unpublished trials were not sought, so publication bias cannot be excluded. Because the evidence base is not pooled quantitatively, the thresholds presented in Table 5 should be read as pragmatic, guideline-anchored operating points rather than as validated decision rules. Reference ranges, assay platforms, and guideline wording vary across institutions and evolve over time, and several cited recommendations will be superseded by future updates. The scope restriction to elective, curative-intent resection is a further limitation: the emergency and post-neoadjuvant pathways are summarized in Section 3.5 but are not reviewed with the same depth, and the review does not address preoperative assessment for cytoreductive surgery, metastasectomy, or re-operative pelvic surgery. Priorities for future work include the standardization and prospective validation of ctDNA-guided perioperative decision-making, which the negative non-inferiority result of DYNAMIC-III shows to be an unresolved question rather than an imminent standard [85,86,87], the definition of the optimal content, intensity, and timing of prehabilitation [72] following its removal as a specific ERAS recommendation [55], the derivation of an internationally agreed skeletal-muscle-index threshold at which a defined intervention is triggered [56,57,58], and the prospective integration of composite biomarker and body-composition data into individualized risk models. The overarching aim is to convert an expanding menu of tests into fewer, better-targeted decisions. Finally, several recommendations offered here are pragmatic syntheses rather than validated algorithms and each requires prospective evaluation: the use of systemic inflammatory indices to triage patients to structured optimization, the nutritional and resistance-exercise interventions triggered by a low skeletal muscle index, and the combination of a Caprini score with disease- and operation-specific features to select candidates for extended thromboprophylaxis. The relevant risk-assessment models also remain imperfect: the Caprini score discriminates only modestly in colorectal cancer surgery and the disease-specific CRC-VTE score requires external validation before it can be recommended in preference [18].

## 9. Conclusions

Preoperative testing before colorectal cancer resection is most valuable when it is purposeful, stage- and risk-adapted, and integrated into a structured perioperative pathway such as the 2025 ERAS Society recommendations [55]. A disciplined core, comprising complete colonic evaluation, appropriate cross-sectional and, for rectal cancer, magnetic resonance staging, a focused laboratory panel with baseline CEA, and widely implemented MMR/MSI testing performed on the diagnostic biopsy, answers the questions that most often change management; in deficient-MMR tumors, that answer may itself redefine whether and how the patient is operated upon. Around this core, the recognition and correction of anemia, malnutrition, CT-defined low skeletal muscle mass, frailty, and hyperglycemia convert measurement into meaningful optimization, provided the deficit is identified early enough for the intervention to work. Emerging biomarkers, led by circulating tumor DNA, are powerfully prognostic and may in time sharpen prognostication and individualize therapy, but current guidelines do not support ctDNA-guided treatment decisions outside clinical trials, and no ctDNA result should currently alter a preoperative plan [87,88]. What the clinician must remember is not a longer list of tests, but a clearer principle: each investigation earns its place only if it refines the stage, changes the plan, or improves the patient before the incision is made. Two operating principles make this practicable: report a prognostic index only when it will be acted upon, and pair every abnormal result with the intervention and the timeframe that it triggers.

## Figures and Tables

**Table 1:** Core preoperative laboratory tests before colorectal cancer resection. TSAT, transferrin saturation; LFT, liver function test; CEA, carcinoembryonic antigen; VTE, venous thromboembolism; CRP, C-reactive protein.

Test	Principal Preoperative Rationale	Clinical Significance, Evidence, and Threshold
Complete blood count	Detect anemia, thrombocytopenia, leukocytosis	Preoperative anemia independently predicts mortality/morbidity and worse cancer survival [7,8]. Anemia: Hb < 130 g/L (men), <120 g/L (women) [9].
Iron studies (ferritin, TSAT)	Characterize iron-deficiency anemia; guide repletion	Frequently underdiagnosed; prognostic in stage II-III disease [10,11]. Absolute deficiency: ferritin < 30 µg/L; functional: ferritin < 100 µg/L with TSAT < 20% or CRP > 5 mg/L [9].
Metabolic panel/LFTs	Baseline organ function; hepatic metastasis clues	Informs hepatic reserve and contrast/drug safety [12].
Coagulation (selective)	Only if bleeding history, liver disease, anticoagulants	Routine screening not recommended; VTE risk dominates [5,13]. Document VTE risk profile preoperatively to set prophylaxis duration [14,15,16]. Stratify with the Caprini score (5 or more = high risk; 9 or more = 30 days) recognizing its modest discrimination in CRC surgery; the Khorana score does not apply postoperatively [17,18,19,20].
CEA	Baseline for surveillance; prognosis	Elevated CEA (>5 ng/mL) independently predicts recurrence/survival [21,22]. Must be drawn before resection and, ideally, before transfusion.

**Table 2:** Diagnostic and staging modalities before colorectal cancer resection. MRF, mesorectal fascia; EMVI, extramural vascular invasion.

Modality	Primary Preoperative Indication	Strengths and Limitations
Colonoscopy + biopsy	Histological diagnosis; synchronous lesions	Strongly recommended; incomplete in obstruction [32].
CT colonography	Colonic completion when scope incomplete	Localizes tumor, finds synchronous lesions [33,34].
Contrast CT chest/abdomen/pelvis	Distant staging (colon and rectal)	Excellent for metastases; modest for local T/N [35,36]. Retained even in the emergency setting [Section 3.5].
Pelvic MRI	Local staging of rectal cancer (reference standard)	Defines MRF, T/N, EMVI; guides neoadjuvant therapy [38,39]. Repeat for restaging after neoadjuvant therapy.
Endorectal ultrasound	Early rectal tumors; complement to MRI	Comparable T-staging accuracy; best for T1–T2 [41].
FDG PET/CT	Problem-solving; equivocal or metastatic disease	Not routine; selective incremental value [42,43].

**Table 4:** Molecular and biomarker testing before colorectal cancer resection, distinguishing established from emerging assays. MRD, molecular residual disease; NLR, neutrophil-to-lymphocyte ratio; PLR, platelet-to-lymphocyte ratio; mGPS, modified Glasgow Prognostic Score; PNI, prognostic nutritional index; MMR, mismatch repair; MSI, microsatellite instability; ctDNA, circulating tumor DNA.

Biomarker	Preoperative/Peri-Diagnostic Role	Evidence Maturity and Current Guideline Position
MMR/MSI (established, not emerging)	Prognosis; eligibility for neoadjuvant and adjuvant immunotherapy; Lynch syndrome screening	Established: widely implemented testing adopted by NCCN, ESMO, ASCRS and ASCP/CAP/AMP/ASCO; ideally on the diagnostic biopsy [81,82,83,84]. Standard of care for every patient; a resection without a documented result is a deviation from standard care.
ctDNA (MRD)	Prognostic after resection; no established preoperative role	Emerging: RCT and large cohorts confirm prognostic value, but DYNAMIC-III did not meet non-inferiority for ctDNA-guided de-escalation, and NCCN/ASCO advise against routine use or treatment decisions outside clinical trials [85,86,87,88].
NLR/PLR	Host inflammatory prognostic index	Emerging: consistent meta-analytic association; no validated threshold for intervention [89,90]. Action: compute, do not order; use to escalate to structured optimization [79,80].
mGPS (CRP/albumin)	Combined inflammatory–nutritional score	Emerging: validated in >9000 patients; prognostic only [91]. Action: mGPS 1–2 triggers nutritional, body-composition and frailty assessment [79,80].
PNI (Onodera)	Nutritional-immune prognostic index	Emerging: independent prognostic value [92]. Action: PNI < 45 triggers dietetic referral and prehabilitation priority [80].
KRAS/NRAS/BRAF	Systemic therapy selection; prognosis	Established for systemic therapy selection in advanced disease; prognostic in localized disease [93,94].

**Table 5:** Practical preoperative checklist for colorectal cancer resection, with thresholds, interventions, timing, and the strength and quality of the supporting evidence. Strength of recommendation and quality of evidence are reported as graded by the cited society guideline (GRADE terminology where available); where a marker is prognostic only and no intervention threshold has been validated, this is stated explicitly. CBC, complete blood count; LFT, liver function test; eGFR, estimated glomerular filtration rate; CEA, carcinoembryonic antigen; Hb, hemoglobin; TSAT, transferrin saturation; CRP, C-reactive protein; NRS-2002, Nutritional Risk Screening 2002; BMI, body mass index; mFI, modified frailty index; CPET, cardiopulmonary exercise testing; HbA1c, glycated hemoglobin; LMWH, low-molecular-weight heparin; VTE, venous thromboembolism; MMR, mismatch repair; MSI, microsatellite instability; ERAS, Enhanced Recovery After Surgery.

Step	Threshold or Trigger	Recommended Action or Intervention	Timing	Strength of Recommendation/Quality of Evidence
1. Confirm and complete	Histology not yet obtained; caecum not reached at colonoscopy	Biopsy confirmation; CT colonography (or early postoperative colonoscopy) to complete colonic evaluation	Before the operative decision; residual colon within 3–6 months if deferred	Strong/moderate [32,33,34]
2. Stage appropriately	Every rectal tumor; equivocal or potentially resectable metastatic findings	Contrast-enhanced CT chest/abdomen/pelvis for all; high-resolution pelvic MRI for all rectal cancers; FDG PET/CT only to resolve a specific question; restaging MRI and CT after neoadjuvant therapy	Within 4 weeks of surgery; restaging 6–8 weeks after completion of neoadjuvant therapy	Strong/high [27,28,35,36,38,39,42,43].
3. Baseline bloods	Coagulation only if bleeding history, liver disease or anticoagulant use	CBC, basic metabolic panel, LFTs, eGFR, glucose and baseline CEA; selective coagulation screen	At diagnosis, and repeated within 4 weeks of surgery if neoadjuvant therapy intervened	Strong/moderate; CEA strong [5,6,30,31].
4. Detect and correct anemia	Hb < 130 g/L (men) or <120 g/L (women); ferritin < 30 µg/L, or <100 µg/L with TSAT < 20% or CRP > 5 mg/L	Intravenous ferric carboxymaltose (approximately 1000–1500 mg total) when surgery is <6 weeks away; oral iron 40–60 mg daily or 80–100 mg alternate days if >6 weeks; restrictive transfusion; erythropoiesis-stimulating agents not routinely recommended	At least 2–4 weeks before surgery (reticulocytosis by day 3–5; measurable Hb rise by 2 weeks)	Strong/high for screening and treatment; moderate for transfusion reduction [9,44,45,46,47,55].
5. Assess nutrition and muscle	NRS-2002 ≥ 3, weight loss > 10% (or >5% in 3 months), BMI < 18.5 kg/m^2^, albumin < 30 g/L; low L3 skeletal muscle index (Prado or Martin cutoffs)	Oral nutritional supplementation, with immunonutrition reserved for malnourished patients; dietitian referral; consider deferring non-urgent surgery in severe nutritional risk; resistance-based exercise where muscle mass is low. Where the skeletal muscle index is low, combine protein 1.2–1.5 g/kg/day with supervised resistance exercise for 4 weeks or more and re-measure on the restaging CT [64,65].	Nutritional support for 7–14 days before surgery	Strong/moderate for nutritional support; moderate for immunonutrition; prognostic only (no intervention threshold) for low muscle mass [50,51,52,53,54,55,56,57,58]. Conditional/low-to-moderate for muscle-targeted intervention [64,65].
6. Gauge fitness and frailty	Age ≥ 70 years, mFI ≥ 2, or subjectively poor exercise tolerance	Objective assessment (CPET, or shuttle/6 min walk test where CPET is unavailable); geriatric or anesthetic review; selective multimodal prehabilitation for the deconditioned or frail	Assessment at the preoperative visit; prehabilitation for 4 weeks where the interval permits	Strong/moderate for frailty and fitness assessment; conditional for prehabilitation (no specific ERAS 2025 recommendation) [55,66,67,68,69,70,72,73].
7. Optimize glycemia and mitigate VTE	HbA1c ≥ 8.5% (69 mmol/mol), or ≥6.5% in a patient not known to be diabetic; day-of-surgery glucose > 10 mmol/L (180 mg/dL); high-risk abdominopelvic cancer surgery Caprini score 5 or more with at least one high-risk feature (advanced stage, open or prolonged pelvic surgery, age > 70 years, previous VTE or thrombophilia, obesity, reduced mobility, raised D-dimer), or Caprini 9 or more	Diabetes team referral and glycemic optimization; treat rather than observe day-of-surgery hyperglycemia; plan pharmacological prophylaxis from admission and extended LMWH for up to 28 days after discharge in high-risk patients, with counseling and self-injection teaching preoperatively	Glycemic optimization 4–12 weeks before surgery; VTE plan agreed at the preoperative visit	Strong/moderate for glycemic control; strong/moderate for extended prophylaxis [6,14,15,16,75]. Extended prophylaxis: strong/high (OR 0.20 for 30-day VTE) [26]; Caprini as a prompt rather than a rule, given modest discrimination in CRC surgery [18].
8. Secure molecular status	Every newly diagnosed colorectal cancer	MMR immunohistochemistry or MSI testing on the diagnostic biopsy; reflex MLH1 promoter methylation and BRAF V600E testing; referral for germline testing and genetic counseling if deficient MMR; RAS/BRAF profiling as the multidisciplinary plan requires; consider neoadjuvant immunotherapy or a trial in deficient-MMR disease	Result available before the multidisciplinary meeting that precedes any neoadjuvant decision	Strong/high (widely implemented) [81,82,83,84,95].
9. Integrate into ERAS	Patients undergoing elective resection	Structured preadmission education and counseling; formal risk and comorbidity assessment; avoid prolonged fasting (solids until 6 h, clear fluids until 2 h); preoperative carbohydrate loading unless contraindicated; document the whole plan in an ERAS pathway	From the first outpatient contact through to admission	Strong/moderate to high [55,74,98,99].

**Table 6:** Convergence of major guidelines on the preoperative workup for colorectal cancer, including the 2025 ERAS Society recommendations. Entries summarize the general direction of recommendations; readers should consult the current version of each guideline for exact wording and evidence grading.

Preoperative Element	NCCN	ESMO	ASCRS/ERAS (2025)
Complete colonoscopy + biopsy	Recommended	Recommended	Recommended
Baseline CEA	Recommended	Recommended	Recommended
CT chest/abdomen/pelvis	Recommended	Recommended	Recommended
Pelvic MRI (rectal)	Recommended	Recommended (standard)	Recommended
MMR/MSI testing	Widely implemented	Widely implemented	Endorsed
PET/CT	Selective	Selective	Not routine
Anemia optimization/counseling	--	Recommended	ERAS 2025 core (strong recommendation)
Nutritional screening/immunonutrition	Recommended	Recommended (ESPEN)	ERAS 2025: screen all; immunonutrition if malnourished
Prehabilitation	Encouraged	Encouraged	ERAS 2025: no specific recommendation
ctDNA/molecular residual disease	Not recommended outside clinical trials	Not recommended outside clinical trials	Not addressed

## Data Availability

No new data were created or analyzed in this study. Data sharing is not applicable to this article.

## Abbreviations

The following abbreviations are used in this manuscript:

ASCO	American Society of Clinical Oncology
ASCRS	American Society of Colon and Rectal Surgeons
BMI	body mass index
CBC	complete blood count
CEA	carcinoembryonic antigen
CPET	cardiopulmonary exercise testing
CRC	colorectal cancer
CRC-VTE	colorectal cancer venous thromboembolism score
CRP	C-reactive protein
CT	computed tomography
ctDNA	circulating tumor DNA
dMMR	deficient mismatch repair
DOAC	direct oral anticoagulant
eGFR	estimated glomerular filtration rate
EMVI	extramural vascular invasion
ERAS	Enhanced Recovery After Surgery
ESPEN	European Society for Clinical Nutrition and Metabolism
EWGSOP2	European Working Group on Sarcopenia in Older People 2
ESMO	European Society for Medical Oncology
FDG PET/CT	fluorodeoxyglucose positron emission tomography/computed tomography
Hb	hemoglobin
HbA1c	glycated hemoglobin
IDA	iron-deficiency anemia
LMWH	low-molecular-weight heparin
LFT	liver function test
mFI	modified frailty index
mFI-5	five-item modified frailty index
mGPS	modified Glasgow Prognostic Score
MMR	mismatch repair
MRD	molecular residual disease
MRI	magnetic resonance imaging
MRF	mesorectal fascia
MSI	microsatellite instability
MSI-H	microsatellite instability-high
NCCN	National Comprehensive Cancer Network
NICE	National Institute for Health and Care Excellence
NLR	neutrophil-to-lymphocyte ratio
NRS-2002	Nutritional Risk Screening 2002
PBM	patient blood management
PLR	platelet-to-lymphocyte ratio
PNI	prognostic nutritional index
RAM	risk assessment model
SMI	skeletal muscle index
SSI	surgical site infection
TSAT	transferrin saturation
VTE	venous thromboembolism
